# siRNA Targeting the 2A^pro^ Genomic Region Prevents Enterovirus 71 Replication *In Vitro*

**DOI:** 10.1371/journal.pone.0149470

**Published:** 2016-02-17

**Authors:** Haibing Liu, Yanyan Qin, Zhenzhen Kong, Qixiang Shao, Zhaoliang Su, Shengjun Wang, Jianguo Chen

**Affiliations:** 1 Department of Clinical Laboratory, The Affiliated People’s Hospital of Jiangsu University, Zhenjiang, China; 2 Department of Immunology, Institute of Laboratory Medicine, Jiangsu University, Zhenjiang, China; National Taiwan University Hospital, TAIWAN

## Abstract

Enterovirus 71 (EV71) is the most important etiological agent of hand, foot, and mouth disease (HFMD) in young children, which is associated with severe neurological complications and has caused significant mortalities in recent HFMD outbreaks in Asia. However, there is no effective antiviral therapy against EV71. In this study, RNA interference (RNAi) was used as an antiviral strategy to inhibit EV71 replication. Three small interfering RNAs (siRNAs) targeting the 2A^pro^ region of the EV71 genome were designed and synthesized. All the siRNAs were transfected individually into rhabdomyosarcoma (RD) cells, which were then infected with strain EV71-2006-52-9. The cytopathic effects (CPEs) in the infected RD cells, cell viability, viral titer, and viral RNA and protein expression were examined to evaluate the specific viral inhibition by the siRNAs. The results of cytopathogenicity and MTT tests indicated that the RD cells transfected with the three siRNAs showed slight CPEs and significantly high viability. The 50% tissue culture infective dose (TCID_50_) values demonstrated that the viral titer of the groups treated with three siRNAs were lower than those of the control groups. qRT–PCR and western blotting revealed that the levels of viral RNA and protein in the RD cells treated with the three siRNAs were lower than those in the controls. When RD cells transfected with siRNAs were also infected with strain EV71-2008-43-16, the expression of the VP1 protein was significantly inhibited. The levels of interferon α (IFN-α) and IFN-β did not differ significantly in any group. These results suggest that siRNAs targeting the 2A^pro^ region of the EV71 genome exerted antiviral effects in vitro.

## Introduction

Enterovirus 71 (EV 71) is a member of the human *Enterovirus A* species, which is classified in the genus *Enterovirus* within the family *Picornaviridae*[1]. Its genome is a positive single-stranded RNA with a long open reading frame and is about 7.4 kb in length. The virus is nonenveloped, and the viral capsid protein consists of four structural proteins, VP1–VP4[2].

EV71 is one of the main etiological agents of hand, foot, and mouth disease (HFMD), which primarily affects infants and young children (< 6 years of age), mainly manifesting as 3–4 days of fever and vesicular exanthema on the buccal mucosa, hands, feet, and oral mucosa[3]. Severe neurological complications, such as encephalitis, aseptic meningitis, acute flaccid paralysis, and a poliomyelitis-like syndrome, are also caused by EV71[4]. More importantly, some EV71-infected children develop pulmonary edema or cardiopulmonary collapse, which can be fatal[5]. These severe clinical symptoms are extremely different from those of coxsackievirus A16, another etiological agent of HFMD, which only causes a mild form of exanthema[6]. In recent years, large-scale HFMD outbreaks have been caused by acute EV71 infections in Asia[7]. More than 1.4 million children have been infected in China[8]. However, there has been no effective treatment for EV71 infection. Faced with such a serious situation, it is urgent that we develop an effective antiviral strategy.

RNA interference (RNAi) is an evolutionarily conserved posttranscriptional gene-silencing phenomenon mediated by small interfering RNA (siRNA), which was first described in *Caenorhabditis elegans*[9]. The gene-silencing process is initiated by long double-stranded RNA (dsRNA) molecules. The dsRNAs are cleaved into small interfering RNAs (siRNAs) of 21–23 nucleotides by an RNase III-like enzyme known as “Dicer”. The siRNA then induces the formation of the RNA-induced silencing complex (RISC), which base-pairs with homologous mRNA to facilitate its degradation[10, 11]. In recent years, several studies have shown that the RNAi approach is an effective tool with which to counter a wide range of viruses, including coxsackievirus B3, human immunodeficiency virus (HIV), and hepatitis B virus[12–14]. Therefore, it is potentially an excellent therapeutic approach for inhibiting EV71 replication.

In this study, we designed three chemically synthesized siRNAs targeting the 2A^pro^ region of the EV71 genome and tested their capacity to inhibit EV71 replication. The cytopathic effects (CPEs) in infected rhabdomyosarcoma (RD) cells, cell viability, viral titer, and viral RNA and protein expression were examined as indicators of the efficacy of targeted gene silencing by the siRNAs. The results showed that three siRNAs effectively inhibited EV71 replication.

## Materials and Methods

### Cell culture and viral strain

RD cells were purchased from the Type Culture Collection of the Chinese Academy of Sciences, Shanghai, China. They were routinely propagated and maintained in Dulbecco’s modified Eagle’s medium (DMEM; Gibco, Grand Island, NY, USA) supplemented with 10% fetal bovine serum (FBS; Gibco) at 37°C with 5% CO_2_. Strain EV71-2006-52-9 (GenBank accession no. KP266579.1) and strain EV71-2008-43-16 (GenBank accession no. KP266572.1) were provided by the Zhenjiang Center for Disease Control and Prevention (Zhenjiang Jiangsu, PR China). The virus was propagated in RD cells.

### Design and synthesis of siRNAs

Three 25-nt double-stranded siRNAs directed against the 2A^pro^ region of the EV71 genome (GenBank accession no. JN001860.1) were designed and designated siRNA-69, siRNA-294, and siRNA-319. A BLAST search showed that the three siRNAs were specific for the target sequences and that they shared no homology with human genes. A scrambled sequence with the same base composition as siRNA-69 was also designed as the negative control (siRNA-SCS) and shared no significant homology with the target gene. A BLOCK-iT Fluorescent Oligo (Invitrogen Life Technologies, Carlsbad, CA, USA) labeled with fluorescein isothiocyanate (FITC) was synthesized to test the transfection efficiency of the siRNAs. All the siRNAs were synthesized by Invitrogen. The sequences of the siRNAs are listed in Table 1.

**Table 1:** Sequences of the synthesized siRNA molecules and their positions in the EV71 2A^pro^ genomic region.

siRNA	Nucleotide sequence	Genomic position	Target gene
siRNA-69	CACTCACAATGATTGGGCAAATCTT GTGAGTGTTACTAACCCGTTTAGAA	69–93	2A^pro^
siRNA-294	CATGCTCGCACAGGGTCACTCAGAA GTACGAGCGTGTCCCAGTGAGTCTT	294–318	2A^pro^
siRNA-319	CCTGGTGATTGCGGTGGTATCCTTA GGACCACTAACGCCACCATAGGAAT	319–343	2A^pro^

### Transfection and infection

RD cells (5 × 10^4^) were seeded into each well of a 24-well plate without antibiotics and grown at 37°C overnight with 5% CO_2_. The culture medium was replaced with 400 μl of prewarmed reduced-serum medium for another 24 h, so that the cells were 30%–50% confluent at the time of transfection. Before transfection, the appropriate amount of siRNA and Lipofectamine 2000 (Invitrogen Life Technologies) was diluted in 50 μl of Opti-MEM I (Invitrogen Life Technologies). After incubation at room temperature for 5 min, the diluted siRNA was mixed gently with diluted Lipofectamine 2000 and the complexes were incubated for 20 min at room temperature. The siRNA–Lipofectamine 2000 complexes were then added to the cell-containing wells and mixed gently by rocking the plate back and forth. After 6 h, the medium was replaced with DMEM supplemented with 2% FBS. The mock-transfected cells were treated as described above but without the siRNA; the negative control cells were treated with the scrambled siRNA; and the virus-only cells were not treated at all. After 24 h, each well was infected with EV71 at a multiplicity of infection (MOI) of 0.01. A variety of indicators were then evaluated at different time points.

### Transfection efficiency assay

The BLOCK-iT Fluorescent Oligo labeled with FITC was used to test the optimal transfection efficiency of the siRNAs. RD cells were transfected with varying concentrations (40, 60, or 80 nM) of BLOCK-iT Fluorescent Oligo and 2 μl of Lipofectamine 2000, as described above. At 24 h after transfection, the cells were washed twice with phosphate-buffered saline (PBS) and the transfection efficiency was evaluated under an inverted fluorescence microscope. The cells were then collected with trypsin (Gibco) digestion and suspended in 200 μl of PBS. The transfection efficiency was evaluated by detecting the FITC-positive RD cells with flow cytometry.

### Cell viability assay and cytopathogenicity

RD cells were seeded in a 24-well plate and then transfected with 60 nM siRNA. After 24 h, the cells were infected with 0.01 MOI of strain EV71-2006-52-9. At one day postinfection, the cells were trypsinized and resuspended in DMEM. The resuspended cells (100 μl) were transferred to a 96-well plate and grown for another 24 h. To assess the viability of the RD cells transfected with siRNA, 3-(4,5-dimethylthiazol-2-yl)-2,5-diphenyltetrazolium bromide (MTT) assays were performed strictly according to the manufacturer’s instructions of the MTT Cell Proliferation Assay Kit (Beyotime, China). Morphological changes in the RD cells transfected with siRNA were evaluated with inverted microscopy at 48 h postinfection. All assays were performed in triplicate in three independent experiments.

### Viral titer assay

RD cells (1 × 10^4^) were seeded into each well of a 96-well plate and incubated at 37°C overnight with 5% CO_2_, so that the cells were 80% confluent at the time of infection. Virus-containing supernatant was serially diluted 10-fold with reduced-serum DMEM, and the culture medium in the 96-well plate was replaced with 100 μl of diluted supernatant. After incubation at 37°C with 5% CO_2_ for 1 h, the diluted supernatant was replaced with 100 μl of DMEM supplemented with 2% FBS. The cytopathic effect (CPE) was observed every day until the experimental endpoint. The TCID_50_ was calculated with the Karber formula. The assay was performed in triplicate in three independent experiments.

### Real-time reverse transcription (RT)–PCR

The primers for real-time RT–PCR were designed and synthesized by Sangon (Shanghai, China) to amplify the region at nt 136–318 in the 2A^pro^ region of the strain EV71-2006-52-9 genome. This segment was used as the standard substance with which to construct the standard curve. The primer sequences were: forward 5′-ACTGCCCAAGGTTGTGAC-3′ and reverse 5′-TTCTGAGTGACCCTGTGC-3′.

At 24 h postinfection, the viral RNA was extracted with the Easy Pure Viral DNA/RNA Kit (Transgen Biotech, China), according to the manufacturer’s instructions. The RT reaction was performed with the EasyScript First-strand cDNA Synthesis SuperMix (Transgen Biotech) in a 20 μl reaction volume, according to the manufacturer’s instructions. Real-time PCR was performed with the Thermo Scientific DyNAmo ColorFlash SYBR Green qPCR Kit (Thermo Scientific, USA) in a 20 μl reaction volume containing 10 μl of qPCR Master Mix, 8 μl of cDNA, and 1 μl of each primer. The cDNA was subjected to 30 cycles of PCR amplification at 95°C for 30 s, 95°C for 5 s, 53.7°C for 15 s and 72°C for 20 s. Absolute quantitation of the viral RNA was determined with the standard curve.

### SDS-PAGE and western blotting analysis

RD cells grown in a 24-well plate were transfected and infected as described above. To obtain sufficient viral protein, each experimental group was treated in two independent wells. At 36 h postinfection, the cells were harvested and lysed with RIPA lysis buffer (Beyotime) to determine the protein concentrations. Equal amounts of protein were separated electrophoretically in an SDS-polyacrylamide gel (12%), and western blotting was performed with standard procedures. The viral protein was detected with a mouse anti-EV71 VP1 monoclonal antibody (Abcam, USA) at a dilution of 1: 1000, and a mouse anti-β-actin antibody (Abcam) at a dilution of 1: 1000 was used as the internal control. A peroxidase-conjugated rabbit anti-mouse IgG secondary antibody (Abcam, USA) was used at a dilution of 1: 5000. The bound antibody was visualized with ECL chemiluminescent substrate (Beyotime).

### Interferon α (IFN-α) and IFN-β assays

RD cells seeded in a 24-well plate were transfected as described above. After 24 h, the culture supernatants of the cells were harvested and the levels of IFN-α and IFN-β were measured with a Human IFN-α ELISA Kit (Cloud-Clone Corp, USA) and a Human IFN-β ELISA Kit (Cloud-Clone Corp), according to the manufacturer’s instructions. The absorbance values were determined at 490 nm.

### Statistical analysis

The data were expressed as means ± SD. The three experimental groups were compared statistically with a *t* test using the SPSS statistical package. Differences with p values < 0.05 were considered statistically significant.

## Results

### Transfection efficiency

**Fig 1:**
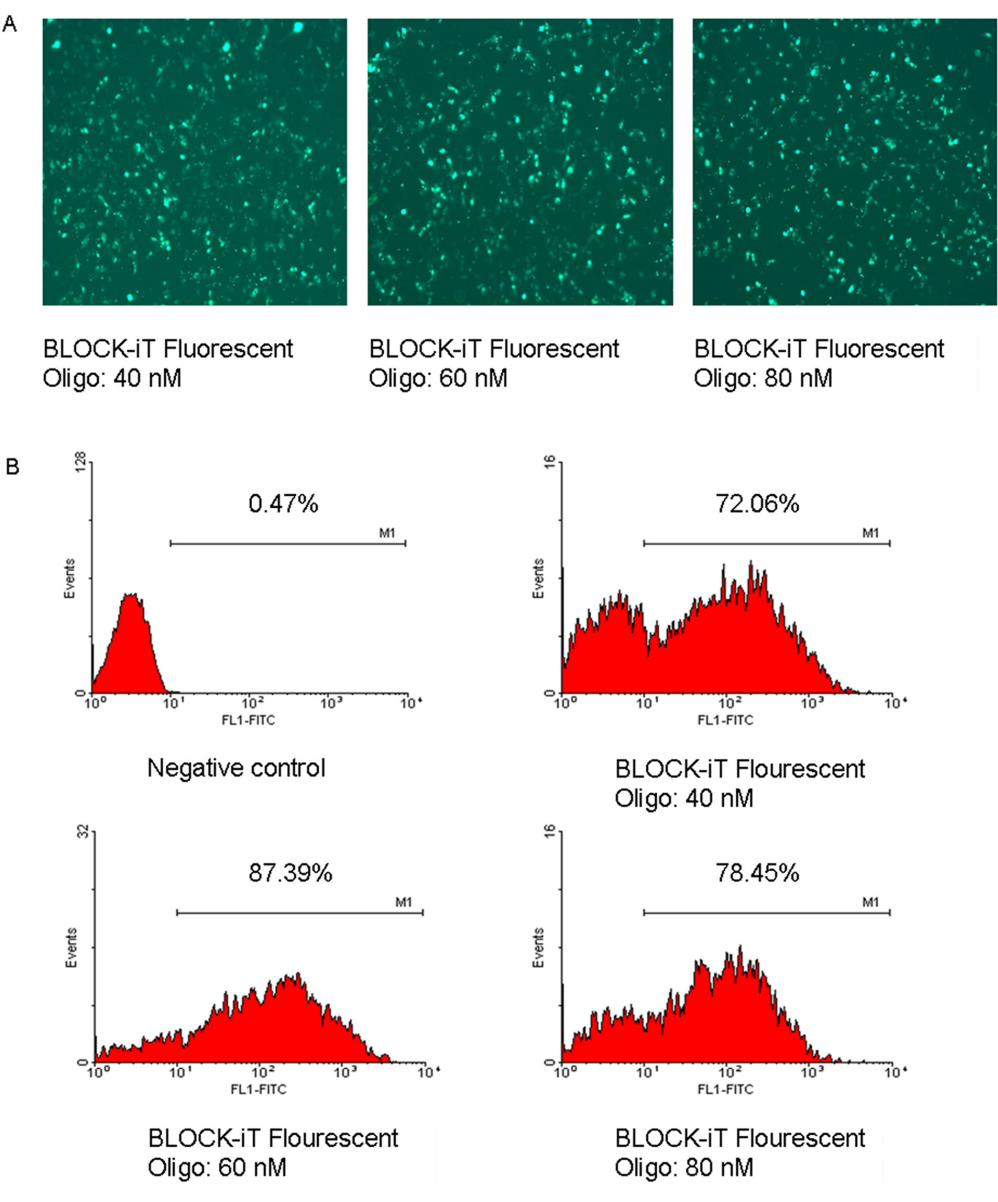
Transfection efficiency of siRNA in RD cells. (A) Cellular distribution of BLOCK-iT Fluorescent Oligo in transfected RD cells. RD cells were transfected with different concentrations of BLOCK-iT Fluorescent Oligo and 2 μl of Lipofectamine 2000. At 24 h after transfection, the cells were observed under a fluorescence microscope. (B) RD cells transfected with BLOCK-iT Fluorescent Oligo were quantified with flow cytometry. The cells were assayed in three independent experiments.

To develop the optimal transfection system, the transfection efficiency of siRNA in RD cells was determined with the BLOCK-iT Fluorescent Oligo labeled with FITC. As shown in Fig 1, the maximal transfection efficiency occurred when the siRNA concentration was 60 nM, when the transfection efficiency was up to 80%. Therefore, the following RNAi experiment was performed with this transfection system.

### siRNAs protect RD cells against EV71-induced cytopathogenicity

**Fig 2:**
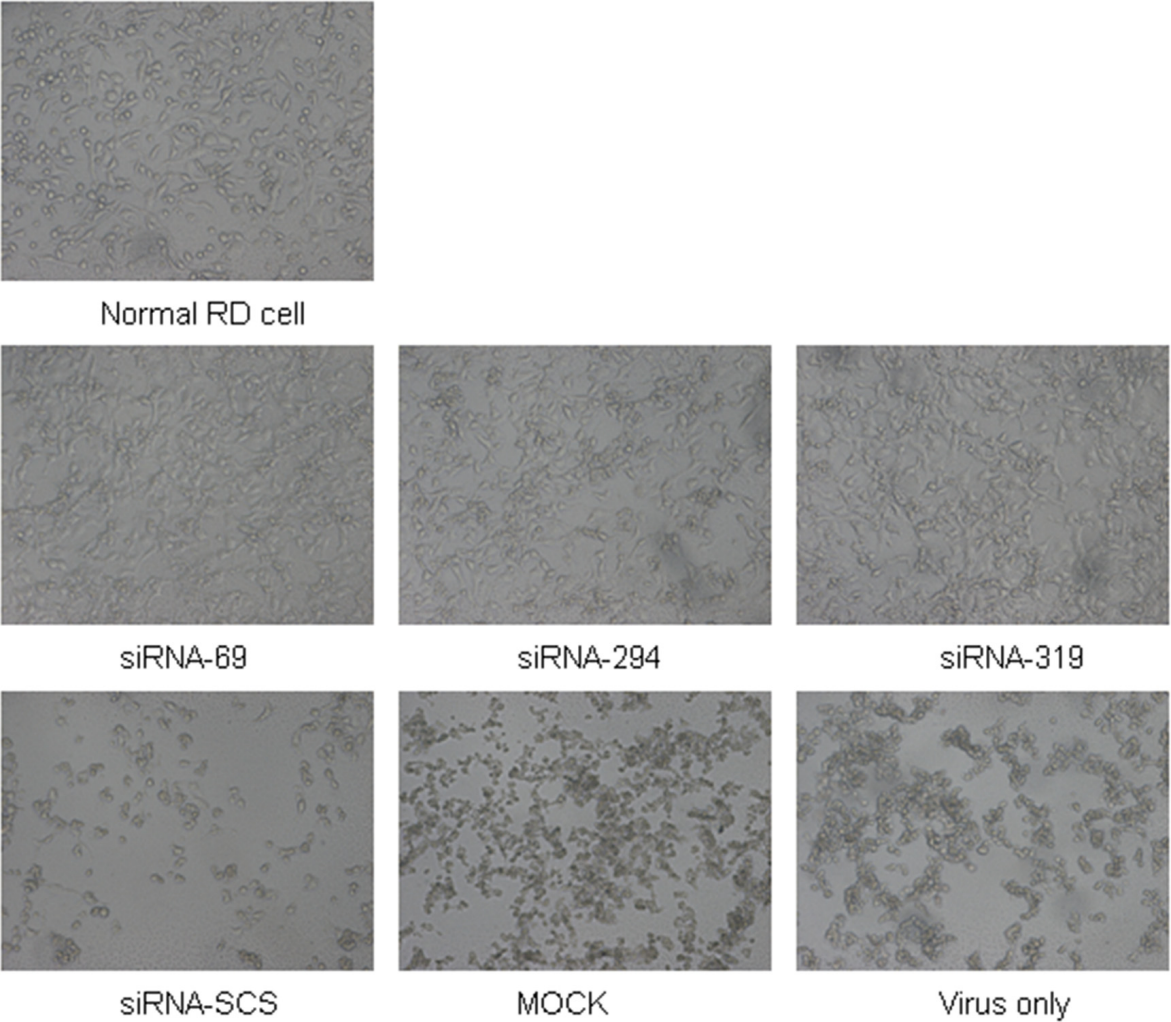
siRNAs protect cells against EV71-induced cytopathic effects. Morphological changes in RD cells were observed after infection. Cells were transfected with each siRNA at a final concentration of 60 nM and then infected with strain EV71-2006-52-9 at an MOI of 0.01. Micrographs were taken at 48 h postinfection under an inverted microscope. The tests were performed in three independent experiments.

To investigate whether the siRNAs protected RD cells from EV71-induced cytopathogenicity, the morphological changes in the RD cells were observed under an inverted microscope. Fig 2 shows that the control groups (cells treated with siRNA-SCS, mock-transfected cells, and cells treated with virus only) displayed significant morphological changes, characterized by cellular condensation, rounding, and nuclear shrinkage at 48 h postinfection. However, the cells transfected with siRNA-69, siRNA-294, or siRNA-319 showed only slight CPEs and higher survival rates. These data confirm that siRNAs directed against the 2A^pro^ region of the EV71 genome successfully protected RD cells from the cytopathogenic effects of strain EV71-2006-52-9 infection.

### Cell viability assay

**Fig 3:**
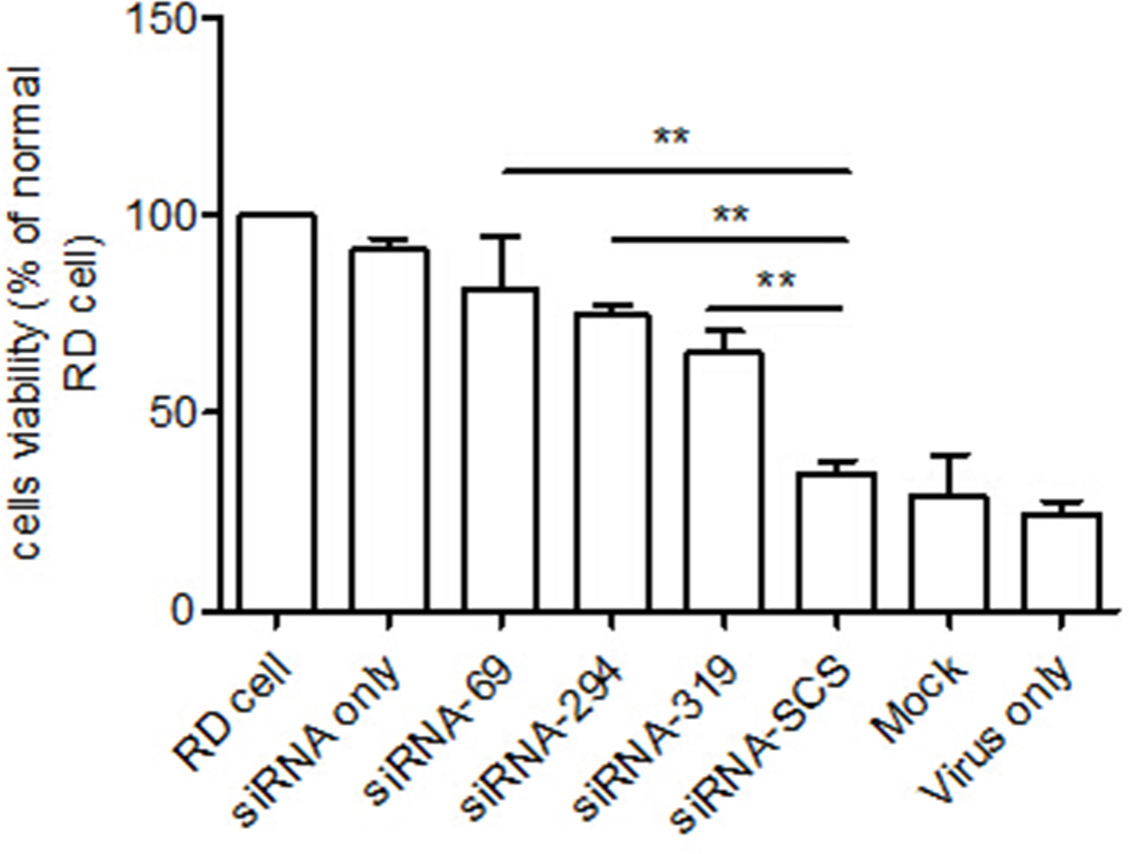
Cell viability assay. Viability was determined with a MTT assay at 48 h postinfection. The cell viability of each group is expressed relative to the normal RD cell control, which was defined as 100% survival. The data shown represent the means ± SD of three independent experiments (**p < 0.01).

An MTT assay was used to confirm the antiviral activities of the siRNAs. The viability of the RD cells transfected with the three siRNAs was significantly higher than that of the control group (Fig 3).

### Viral titer assay

**Fig 4:**
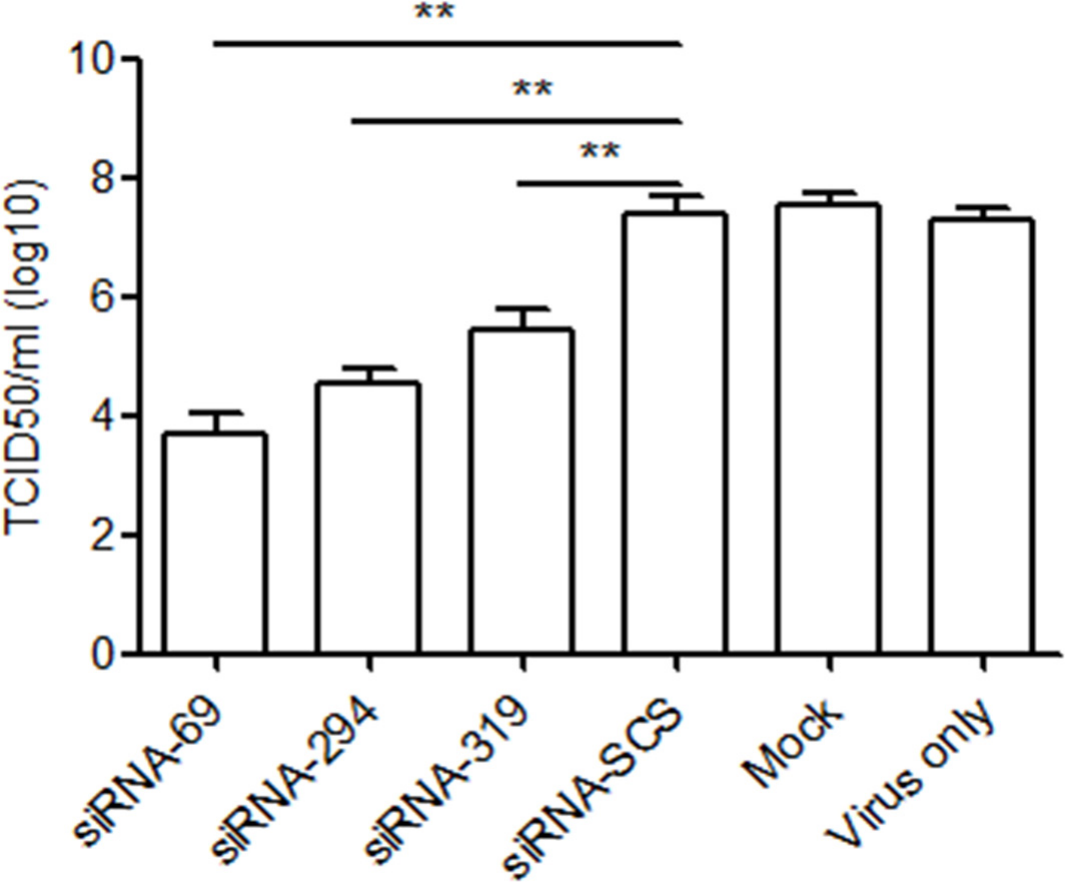
Viral titer assay. Virus titers were determined in term of TCID_50_. RD cells pretreated with siRNAs were infected with strain EV71-2006-52-9 at an MOI of 0.01. At 48 h postinfection, the supernatants were collected to detect the progeny viral titers. Data are the means ± SD of three independent experiments (**p < 0.01).

The viral titer was evaluated (in terms of TCID_50_ values) to further demonstrate the inhibitory effects of the three siRNAs on the replication of strain EV71-2006-52-9. Fig 4 shows that the TCID_50_ values of the groups treated with the three siRNAs were significantly lower than those of the control groups

### siRNAs inhibited EV71-specific RNA and protein expression

**Fig 5:**
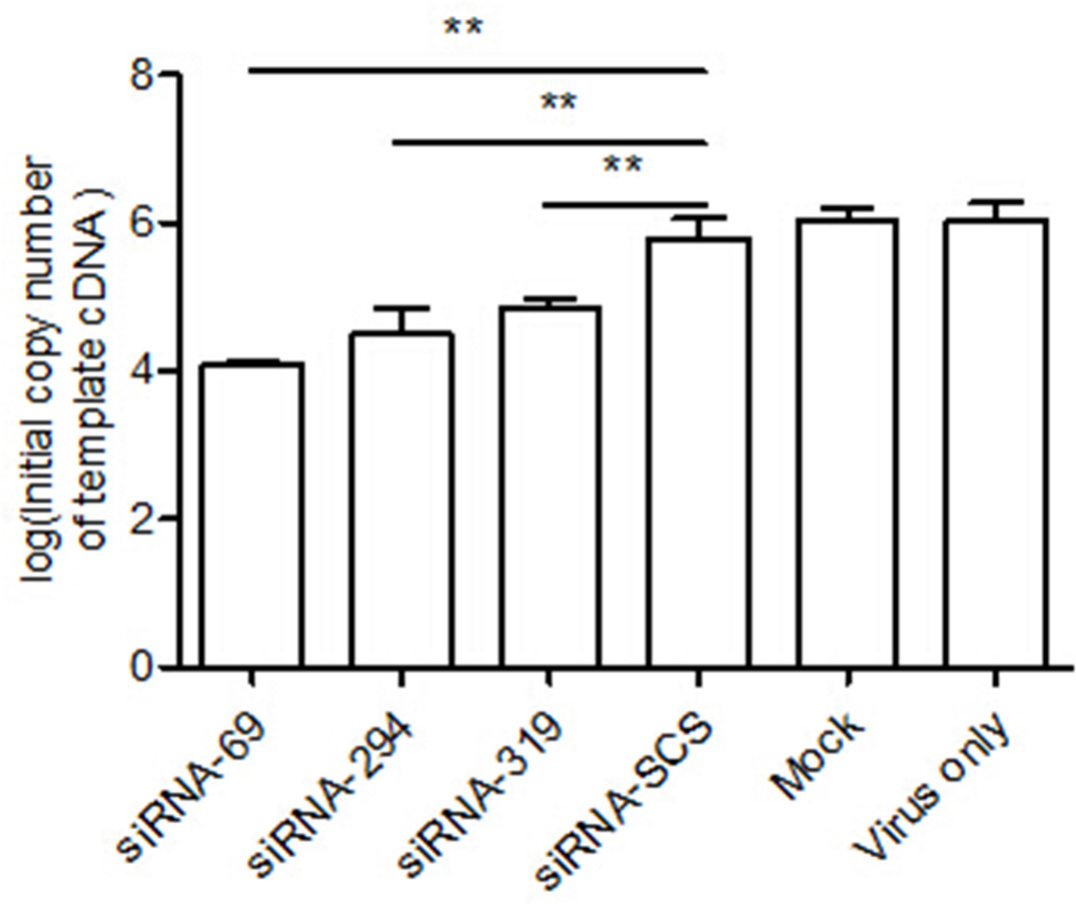
RNAi inhibits viral RNA. RD cells were treated with each siRNA and then infected with strain EV71-2006-52-9 at an MOI of 0.01. At 24 h postinfection, the viral RNA was extracted and analyzed with real-time RT–PCR. The data shown represent the means ± SD of three independent experiments (**p < 0.01).

We next assessed the inhibitory effects of each of the three siRNAs on strain EV71-2006-52-9 replication with real-time RT–PCR and western blotting. The results of qRT–PCR indicated that the levels of viral RNA in the RD cells treated with siRNA-69, siRNA-294, or siRNA-319 were lower than in cells transfected with siRNA-SCS (Fig 5).

**Fig 6:**
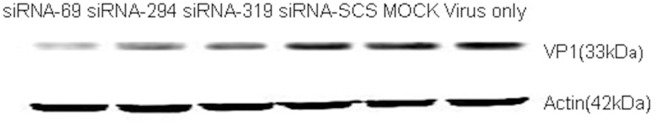
RNAi inhibits viral protein. RD cells transfected with siRNA were infected with strain EV71-2006-52-9 at an MOI of 0.01. At 36 h postinfection, total protein was extracted and analyzed with western blotting. β-Actin was used as the internal loading control. The protein measurements were made in three independent experiments.

The expression of specific EV71 viral capsid protein VP1 was measured with western blotting to investigate whether the reduction in specific viral RNA transcripts correlated with the downregulation of specific viral proteins. As shown in Fig 6, strain EV71-2006-52-9 VP1 protein was significantly reduced in the cells treated with the siRNAs targeting the 2A^pro^ region of the EV71 genome, but was reduced only slightly in the control groups.

### RNAi inhibits the replication of different EV71 strains

**Fig 7:**
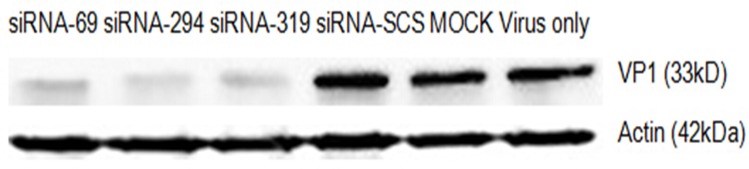
RNAi inhibits the replication of different EV71 strains. RD cells transfected with siRNA were infected with strain EV71-2008-43-16 at an MOI of 0.01. Total protein was extracted at 36 h after infection, and VP1 expression was analyzed with western blotting. β-Actin was used as the internal loading control. Protein measurements were made in three independent experiments.

To verify that the replication of different EV71 strains was inhibited by the three siRNAs, we tested the inhibitory effects of each of the three siRNAs on strain EV71-2008-43-16 with western blotting. VP1 protein expression decreased dramatically in the cells treated with siRNA-69, 294 and 319 (Fig 7).

### IFN pathway was not activated in RD cells treated with siRNAs

**Fig 8:**
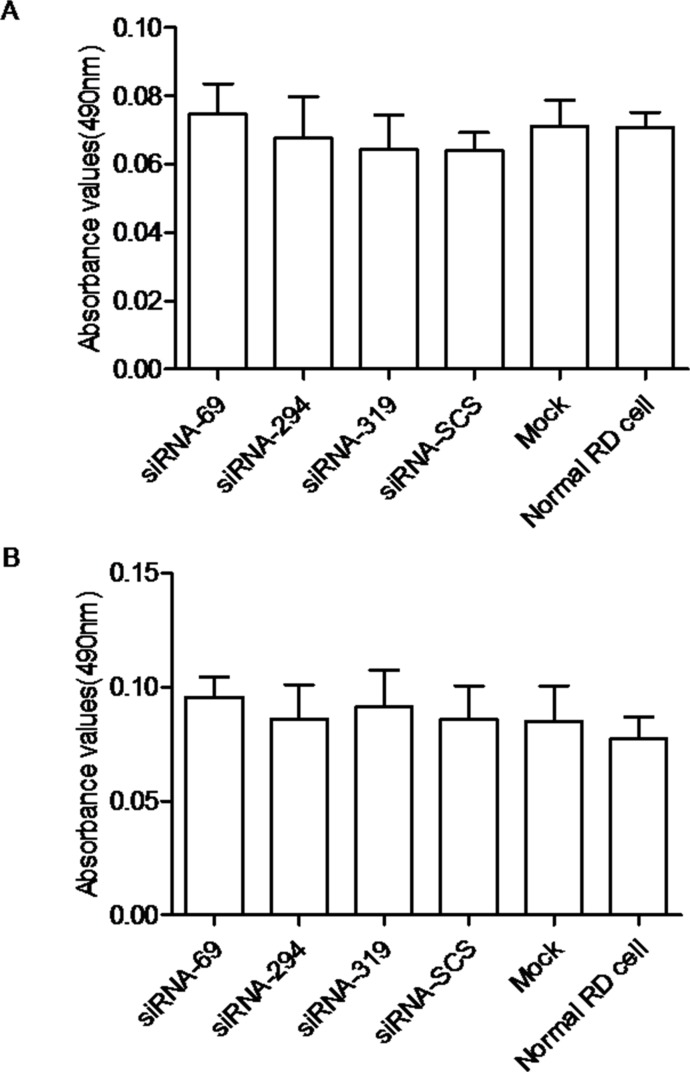
Interferon pathway was not activated by siRNA. The levels of IFN-α (A) and IFN-β (B) were determined by measuring the absorbance at 490 nm. The data shown represent the means ± SD of three independent experiments.

To confirm that EV71 inhibition was attributable to the specific antiviral effects of siRNAs targeting the 2A^pro^ region of the EV71 genome and not to the activation of IFNs, we measured the levels of IFN-α and IFN-β in the culture supernatants of the cells. The results showed that the absorbance did not differ significantly in any group. Therefore, we conclude that the inhibition of EV71 was mediated by RNAi (Fig 8).

## Discussion

In the last few years, the RNAi technique has been used extensively as a potential therapeutic tool for the treatment of many diseases, including cancer, bone-related diseases, and cardiovascular diseases[10]. Great success has also been achieved with RNAi in the treatment of viral diseases, including those caused by polioviruses, HIV, and hepatitis B virus. Different types of siRNAs targeting various regions throughout the entire EV71 genome (VP1, VP2, 5′-UTR, 3′-UTR, 2C, 3C^pro^, and 3D^pol^) are reported to exert varying degrees of antiviral activity[15–18]. However, the use of the 2A^pro^ region of the EV71 genome as an antiviral target has not been reported. Protease 2A^pro^ cleaves both the viral polyprotein and translational factor eIF4GI to shut down the host cell translation[19, 20]. The 2A^pro^ region of the genome is also highly conserved and less likely to mutate than other regions of the EV71 genome[21], which is an important factor in RNAi therapy. Therefore, we chose it as the target when designing siRNAs, to explore whether the 2A^pro^ gene is a potential target for RNAi.

In this study, three siRNAs against the 2A^pro^ region of the EV71 genome were designed and synthesized. We infer from the results that the siRNAs targeting the 2A^pro^ region are highly effective in inhibiting EV71 replication and virus-mediated cell injury or death in vitro. Longer dsRNAs reportedly elicit a nonspecific interferon response, resulting in the expression of large numbers of IFN molecules, leading to the inactivation of cellular transcription and the death of mammalian cells[22, 23]. This IFN-mediated response can inhibit viral replication, so it was important to determine whether these siRNAs activated the IFN response. Our results show that the IFN response was not elicited by these siRNAs, so the inhibition of EV71 replication was specifically caused by the siRNAs, and not by an undesirable side effect.

In conclusion, we have demonstrated that siRNAs targeting the 2A^pro^ region of the EV71 genome inhibit EV71 replication with very high efficiency in vitro, so this highly conserved region of EV71 is a potential target for the inhibition of viral replication by RNAi. In previous studies, chemically synthesized siRNAs have displayed poor antiviral effects in vivo[24, 25], so whether the siRNAs designed in this study inhibit EV71 replication in vivo requires further research.
